# The association between medial prefrontal GABA concentration and memory performance is disrupted in human with a high body mass index

**DOI:** 10.1007/s11682-026-01121-1

**Published:** 2026-03-27

**Authors:** Jan-Willem Thielen, Bernhard W. Müller, Oliver Kraff, Dae In Chang, Constantin Liermann-Koch, Norbert Scherbaum, Indira Tendolkar, David G. Norris

**Affiliations:** 1 Erwin L. Hahn Institute for Magnetic Resonance Imaging, Essen, Germany; 2 Department for Psychiatry and Psychotherapy, Faculty of Medicine, University of Duisburg-Essen, Essen, Germany; 3 Department for Psychiatry, Faculty of Medicine, University of Bochum, Bochum, Germany; 4 Department for Cardiology, Internal intensive Medicine, Medical Clinic Center Dortmund, Dortmund, Germany; 5 Donders Institute for Brain Cognition and Behavior, Radboud University, Radboud University Medical Center, Nijmegen, the Netherlands; 6 Department of Psychiatry, Radboud University Medical Center, Nijmegen, the Netherlands; 7 MIRA Institute for Biomedical Technology and Technical Medicine, University of Twente, Enschede, the Netherlands

## Abstract

While the somatic effects of obesity are increasingly well understood, growing evidence indicates that there is a cognitive effect such as a decline in episodic memory. Studies in animals revealed an association between obesity, memory impairment and neurotransmitters such as γ-aminobutyric acid (GABA) and glutamate. In the present study we aimed to assess the relation of body mass index (BMI) as indicator of overwight/obesity, memory and GABA / glutamate concentrations in the medial prefrontal cortex (mPFC) and precuneus in human subjects aged 19 to 41 years. We found that memory performance is predicted by mPFC GABA concentration in individuals with a low and a moderate BMI, but not in individuals with a high BMI (> 26) indicating overweight/obesity. MPFC glutamate and precuneus GABA/ glutamate levels appeared to be not related to the association between BMI and memory performance. Our findings suggest that clinically relevant overweight/obesity affects GABAergic processes in the frontal cortex and is related to lower memory performance in a group of young and middle-aged human subjects.

**Supplementary Information:**

The online version contains supplementary material available at 10.1007/s11682-026-01121-1.

## Introduction

Obesity is a serious and growing public health issue that decreases life expectancy due to its association with blood hypertension, cardiovascular disease, glucose intolerance, hyperinsulinemia and type 2 diabetes (Kaur, 2014). While the somatic effects of obesity on physical health are increasingly well understood, recent research indicates that there are noteworthy cognitive effects related to obesity (Zhang et al., 2022; Cheke et al., 2016). It has been shown that obesity, as measured with BMI, is inversely associated with performance in neurocognitive tests of attention and executive function (Lentoor and Myburgh, 2022). Moreover, there is evidence that obesity is associated with reduced episodic memory performance (Cheke et al., 2016) and the occurrence of mild cognitive impairment, a disease state premorbid to dementia (Michaud et al. 2018). The capability to remember past experiences is important and a decrease of this ability causes impairment in quality of life, in activities of daily living and for the government immense cost in the realm of care giving (Marešová et al., 2020). Therefore, understanding the mechanisms of the association between obesity and lower memory performance are important.

One possible mechanism behind the association between obesity and memory performance may be related to the brain’s main inhibitory neurotransmitter γ-aminobutyric acid (GABA) and/or the excitatory neurotransmitter glutamate. Recent animal studies revealed associations between obesity and neurotransmitter functioning. For instance, it has been shown that high fat diet (HFD) decreases GABA levels in the frontal cortex and hippocampus of rats (Sandoval‑Salazar et al., 2016). Conversely, other studies could not report effects of HFD on GABA levels in the hippocampus despite an association between HFD and memory performance. For instance, Martínez-Orozco and colleagues (2021) showed that HFD and a combined high-fat–high-fructose diet impaired memory functioning in mice without an effect on hippocampal GABA concentration.

In humans, obesity is associated with many diseases, most notably type 2 diabetes (Algoblan et al., 2014). In this regard we have previously shown in a Magnetic Resonance Spectroscopy (MRS) study that patients suffering from type 2 diabetes revealed increased GABA concentrations in the medial prefrontal cortex which were then negatively associated with episodic memory performance (Thielen et al., 2019). Our result is in line with pharmacological studies showing that GABA receptor antagonists enhance memory consolidation and its agonists impair memory function (McGaugh and Roozendaal, 2008). Obesity appears not only to be associated with the inhibitory neurotransmitter GABA but also with the excitatory neurotransmitter glutamate. Martínez-Orozco and colleagues (2021) showed that HFD and a combined high-fat–high-fructose diet in mice decreased glutamate concentrations in the hippocampus which was associated with impaired memory functioning. Another study demonstrated that obese rats showed an increased level of glutamate in whole brain tissue (Labban et al., 2020), which may suggest that obesity may either increase or decrease glutamate concentrations depending on the specific brain region. The connection between glutamate and memory appears to be related to the capacity of NMDA receptors to induce long-term potentiation (LTP); a bioelectric process linked to synaptic plasticity (McEntee and Crook, 1993; Collingridge and Bliss 1987) which is viewed as a cellular model for learning and memory (Nicoll et al. 1988). Similar to the aforementioned GABA MRS Study (Thielen et al., 2019), a another glutamate MRS study by our group revealed an association between mPFC glutamate concentration and memory performance (Thielen et al., 2018). In this study mPFC glutamate/glutamine (GLx) concentrations were measured before and after volunteers memorized face–name associations. We found that mPFC GLx levels increased during the memory task, which appeared to be positively associated with memory performance.

As mentioned above, obesity is associated with many diseases, most notably type 2 diabetes (Algoblan et al., 2014). In obese individuals, the amount of glycerol, nonesterified fatty acids, cytokines, proinflammatory markers, hormones and other substances that are involved in the development of insulin resistance, are increased (Algoblan et al., 2014). Therefore, obesity is considered the most important factor in the development of metabolic diseases such as type 2 diabetes (Algoblan et al., 2014, Karpe et al., 2011). As previously mentioned, we have shown that patients suffering from type 2 diabetes have increased GABA concentrations in the medial prefrontal cortex which are negatively associated with episodic memory performance (Thielen et al., 2019) In the present work, we explore whether this effect is already evident in obese nondiabetic individuals.

Since BMI has a strong relationship to type 2 diabetes and insulin resistance (Zhao et al., 2017; Sinaiko et al., 2005) we performed a moderator analysis with BMI as moderator variable. A moderator analysis is used to determine whether the relationship between two variables depends on (is moderated by) the value of a third variable. In this regard we measured GABA and glutamate/glutamine concentrations in the mPFC and precuneus, and investigated if an association between the neurotransmitter concentrations and memory performance is determined by overweight/obesity as measured with BMI.

## Methods

### Participants

This study was conducted in 65 healthy nondiabetic humans (22 male/43 female; mean age = 24.80 SD = 5.25; mean BDI = 23.34 SD = 3.21). Participants were recruited between 21.06.2017 and 13.11.2020. Inclusion criteria were age between 18 and 45 years and a BMI between 17 and 40 kg/m^2^. None of the subjects used any medication, had a history of neurological or psychiatric illness, drug abuse, or head trauma. The study was approved by the local medical ethics committee and written informed consent was obtained from each subject.

### Experimental design

Before entering the scanner, the subjects were weighed and their height was measured. In the scanner subjects underwent a short structural scan to guide the placement of the MRS voxels. Thereafter, we conducted MRS scans of two voxels positioned in the mPFC and precuneus, which was followed by the study phase of a face profession association task (see below). After scanning, subjects performed a memory test outside the scanner.

### Memory paradigm

The learning phase of the face-profession task was done within the scanner and contained six blocks of an episodic memory condition consisting of four stimuli (faces with their associated occupational titles underneath) which were interleaved with six blocks of a visuo-motor task containing six stimuli (shadow-masked face contours) whereby each block lasted 22.8 s. Each block started with the presentation of a brief instruction for 2.0 s indicating which condition follows. In the memory condition, each face and its correspondent occupational title below was displayed for exact 5.7 s at the center of the screen. Participants had to judge (via a button box) whether the profession and the face fitted well or not and were then instructed to memorize these face-profession associations for a subsequent memory test outside the scanner. In the visuo-motor condition shadow-masked face contours were displayed for exact 3.8 s at the center of the screen. Participants were instructed to judge (via button box) whether the ears of the faces were closer to the right or the left shoulder (Thielen et al., 2016/2019).

**Fig. 1:**
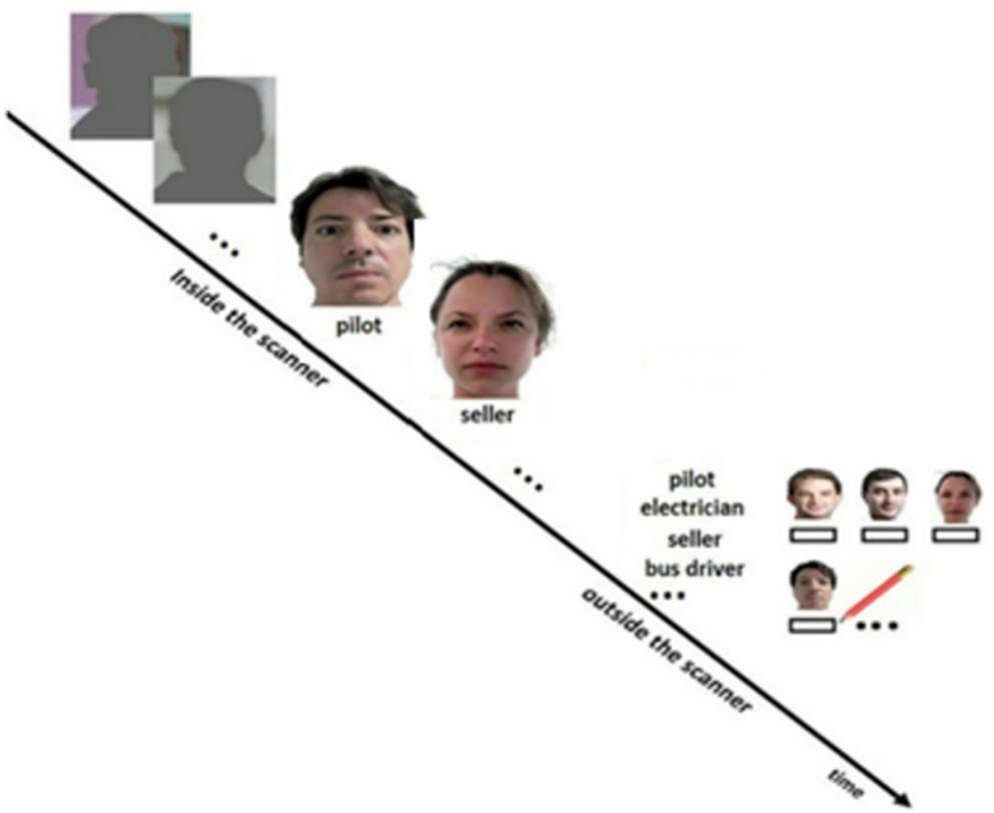
Design of the face-profession association task. Within the scanner, volunteers performed the face-profession association task that was interleaved with the visuo-motor task. In the face-profession association task volunteers had to memorize the face-profession associations and to indicate whether the face fitted well with the profession or not. During the visuo-motor task (Shown in the upper part of the figure), volunteers had to indicate whether the ears of a shadow-masked face contour were nearer to the right or the left shoulder. Subsequently, they performed a recall test outside the scanner. The figure is adapted with permission from Thielen et al. (2016)

After the scanning, the participants performed a recall test for the associated professions. The volunteers were presented with all the faces (printed on papers in A4 format) and had to indicate which professions belonged to the different faces (Fig. 1). Performance was determined by the amount of correct remembered face-profession associations.

### MRS data acquisition

Scanning was conducted on a 7 T MR system (Magnetom 7 T, Siemens Healthcare, Germany) using a single-channel transmit 32-channel receive head coil (Nova Medical, Wilmington, MA). To guide the placement of the MRS voxel, a three-dimensional T1-weighted structural image (MP2RAGE; TR = 2,300 ms, TI = 1,100 ms, TE = 3.03 ms, FOV = 240 × 240 mm, spatial resolution = 1 × 1 × 1 mm^3^, 192 slices, flip angle = 8°) was acquired. Subsequently, single voxel edited ^1^H-MR Spectra from precuneus (voxel size: 20 × 20 × 20 mm^3^) and the medial prefrontal cortex (voxel size: 20 × 20 × 20 mm^3^) were acquired when the B_0_ shimming procedure resulted in a full width at half maximum (FWHM) lower than 30 Hz as a quality criteria for the MRS scans. MEGA-sLASER (TR = 4,500 ms, TE = 80 ms, number of excitations = 64 [32 on, 32 off], F1 = 2500 Hz; 1024 pts; frequency offset − 1.69 ppm [−507 Hz], water suppression method = WET, scan time = 5:06 min) was used as an editing method (Andreychenko et al., 2011). According to Lin and colleagues 2021 there are multiple methods of acquisition, post-processing, and analysis of MRS data. Consequently, the authors recommend MRS reporting standards (Lin et al., 2021). Therefore, we have included the MRSinMRS checklist (Lin et al., 2021) as Supplementary Material.

### MRS voxel placement

As shown in Thielen et al. (2016) the episodic memory task used in the current study activates brain regions in the precuneus and mPFC. Therefore, we placed the MRS voxels in these brain regions. The placement of the voxels was done in accordance with anatomical landmarks.

**Fig. 2:**
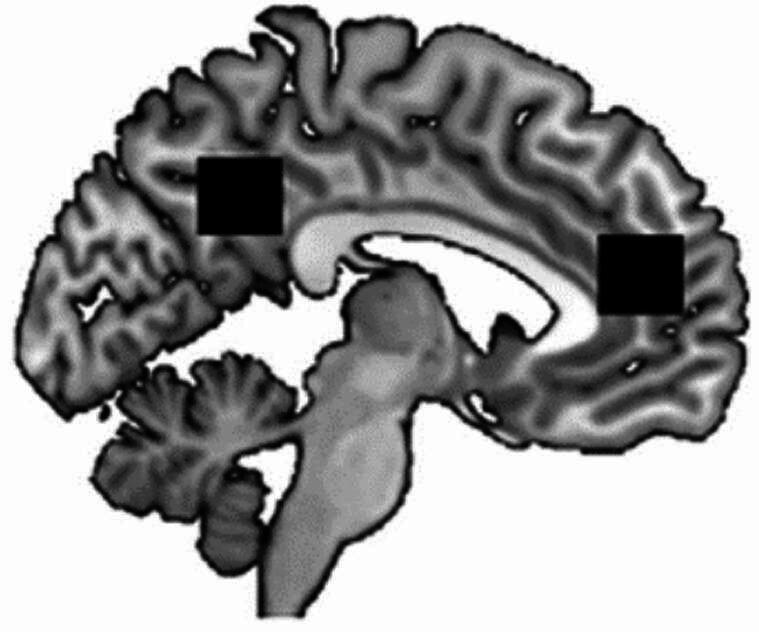
The positions of the MRS voxels (black squares) are illustrated. The right (anterior) voxel is located in the medial prefrontal cortex (mPFC) and the left (posterior) voxel is located in the precuneus (Voxel size: 20 × 20 × 20 mm). The figure is adapted with permission from Thielen et al. (2019)

The mPFC voxel was placed at the anterior edge of the bent of corpus callosum.

and the precuneus voxel was placed between the parieto-occipital fissure and the edge of the posterior bend of corpus callosum (see Fig. 2; Thielen et al. 2019).

### MRS data processing

**Fig. 3:**
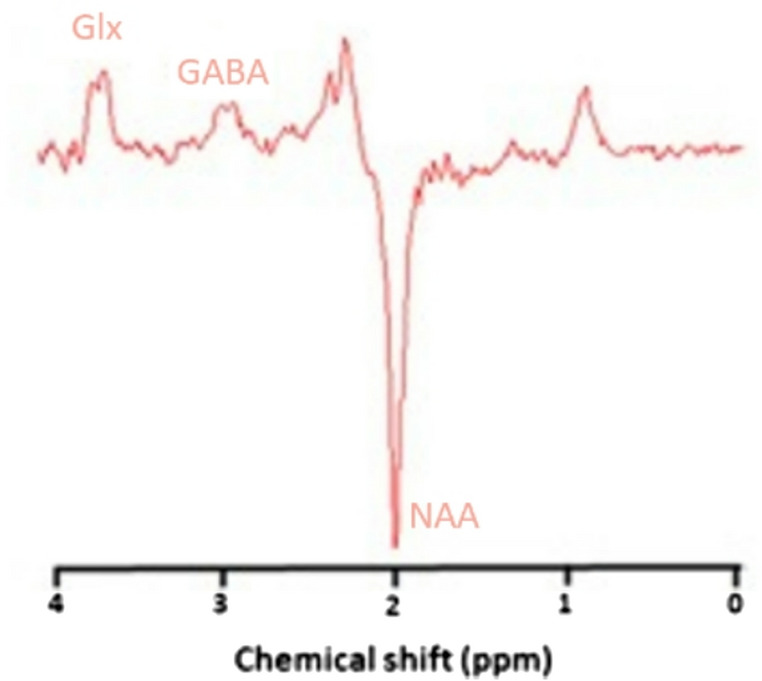
The figure shows the difference spectra („edit off” minus “edit on”) yielding the γ-aminobutyric acid (GABA) and glutamate‐glutamine (Glx) peaks

The difference spectra were calculated by subtracting the edit on and edit off spectra. The ratio of glutamate/glutamine (GLx) and GABA to N-Acetyl-aspartat (NAA) was determined using the AMARES package (Vanhamme et al., 2001) integrated within the jMRUI software (Naressi et al., 2001). Prior to fitting in jMRUI, all spectra were apodized with a 5 Hz Lorenzian filter. Whereas, NAA was modeled from the “edit off spectra” as a single Lorentzian peak, GLx and GABA were modeled from the “different spectra” as a pair of Lorentzian peaks with the same line width as NAA (see Fig. 3 for a representative spectra).

The quality of the data was assessed by using the signal to noise ratio (SNR). The SNR was calculated as the ratio of the peak amplitude of NAA to the standard deviation of the noise, measured in a signal-free region of the spectrum (9–10 ppm). Spectra with SNR values > 20 were considered high quality. Values between 15 and 20 were deemed acceptable but suboptimal. Since we did not make tissue correction the measured GABA and Glx concentration were the sum from the whole MRS voxel with metabolites from gray matter, white matter and cerebrospinal fluid (CSF).

To probe the associations of mPFC/precuneus GABA/GLx levels and memory performance and BMI partial correlation analyses controlled for age and gray matter ratio of the specific MRS voxels were performed.

### Statistical analysis (moderator analysis)

To assess whether BMI has an effect on the association between GABA/GLx and memory performance (see Thielen et al. 2019) we conducted a moderator analysis utilizing the PROCESS macro for SPSS (PROCESS v2.16, Hayes, 2016). We used Model 1 which is a moderation analysis with one continuous moderator variable. GABA or GLX served as independent variable, memory performance as dependent variable and BMI as moderator variable controlled for age, sex and gray matter ratio of the specific MRS voxel (mPFC/precuneus).

## Results

### Characteristics of the sample

**Fig. 4:**
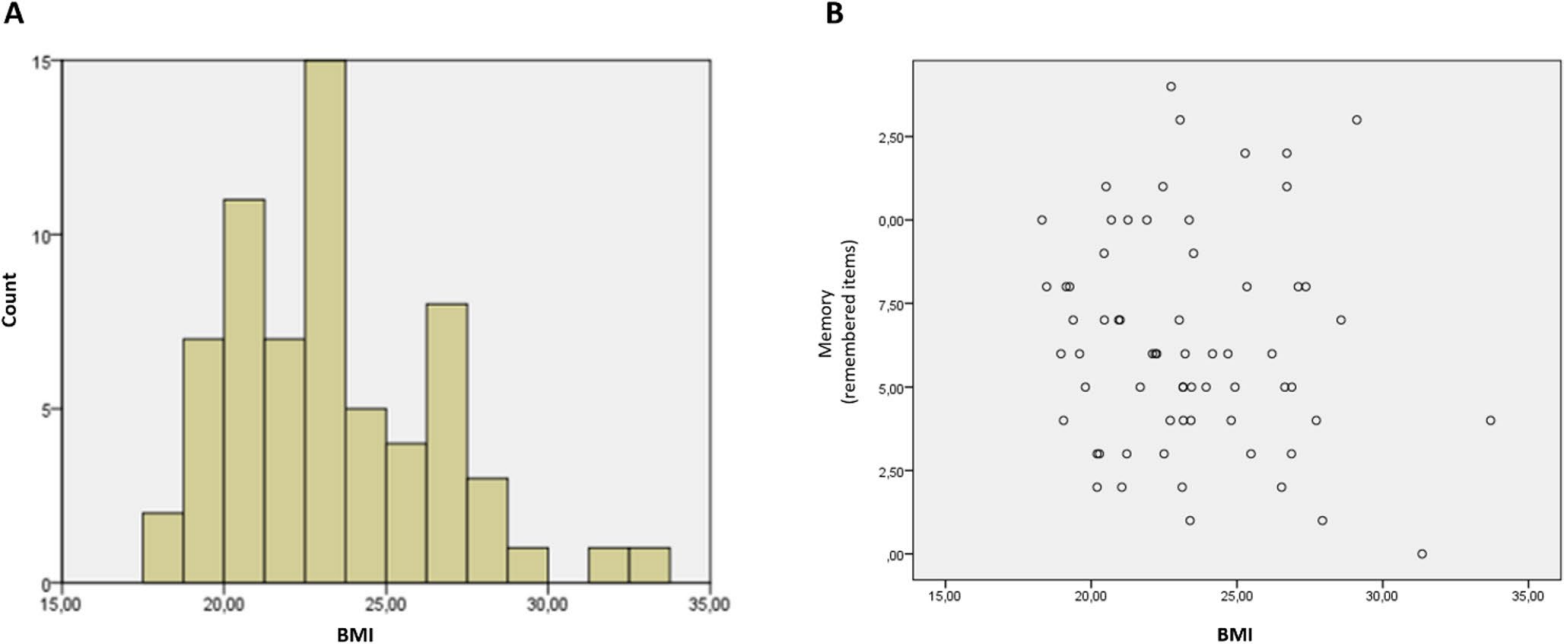
In **A** the distribution of participants in terms of BMI. In **B** the correlation between memory performance and BMI are depicted

In this study we analyzed 65 participants (22 male/43 female; mean age = 24.80 SD = 5.25 years). The sample mean BMI was 23.34 (see Fig. 4) with a minimum BMI of 18.31 and a maximum of 33.70 (SD = 3.21). BMI of male and female participants differed significantly (*p* <.05) with male mean BMI of 24.58 (SD = 3.68) and female mean BMI of 22.71 (SD = 2.77). Moreover, there was a significant sex difference regarding age. Males were significant older than the females (*p* <.02) with a mean age of the males 28.27 years (SD = 6.97) and females 23.02 years (SD = 2.86). Memory performance ranged from 1 to 16 remembered items with a mean of 6.49 items (SD = 3.57).

### Neurotransmitter and memory performance/BMI

**Fig. 5:**
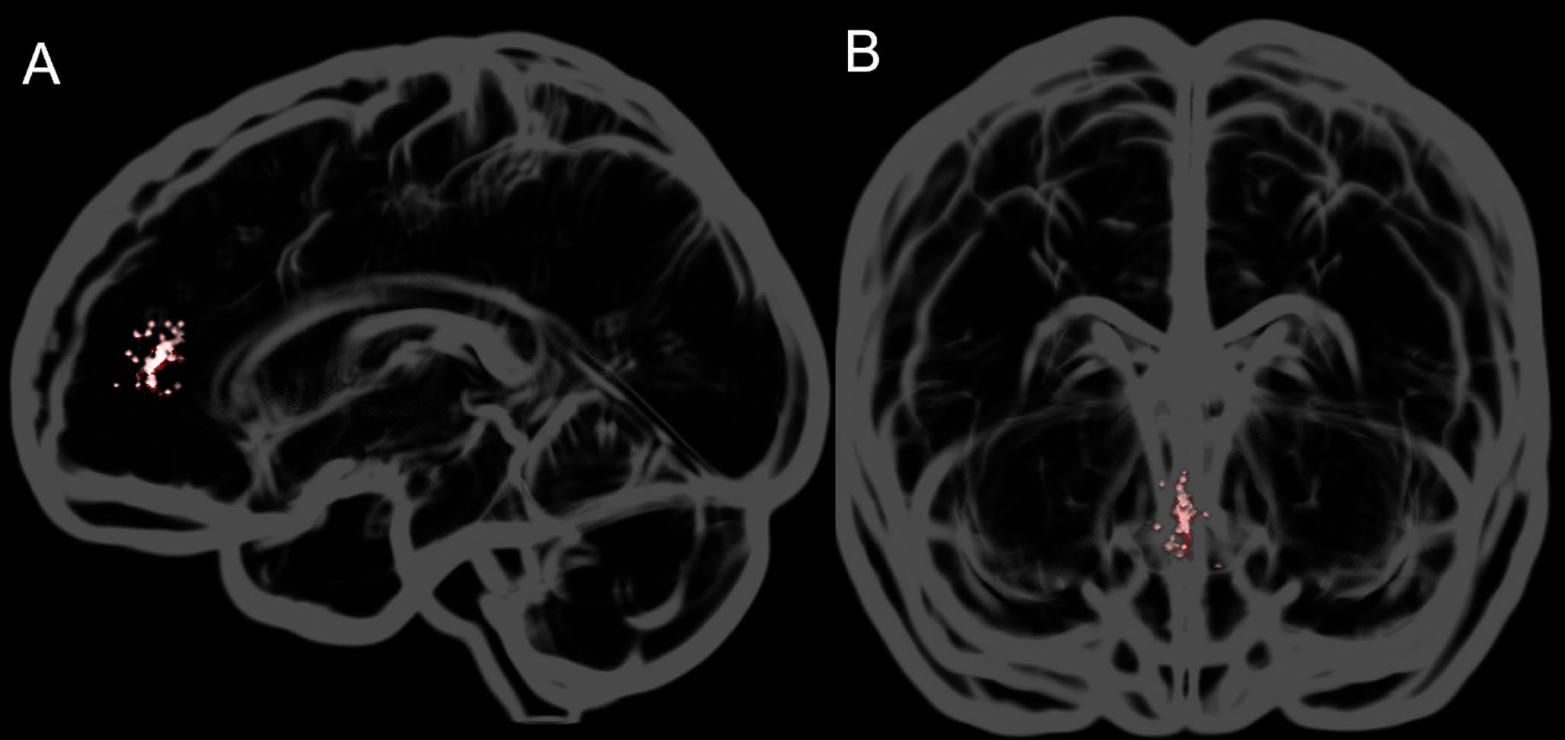
The center of the mPFC MRS voxel for each participant is depicted on the glass brain template included in the MRIcroGL software (Rorden and Brett, 2000). In **A** the centers of mass are shown from a side view **B** shows the centers of mass from the front

The mPFC mean voxel position (center) in MNI space was *x* = − 1, *y* = 44, *z* = 13 with a mean percentage of 17% (*SD* = 0.102) white matter, 72% (*SD* = 0.092) gray matter, and 11% (*SD* = 0.048) CSF. In Fig. 5 the center of the mPFC MRS voxel for each participant is depicted.

The precuneus mean voxel position (center) in MNI space was *x* = − 1, *y* = −46, *z* = 35 with a mean percentage of 18% (*SD* = 0.038) white matter, 71% (*SD* = 0.053) gray matter and 11% (*SD* = 0.052) CSF. First, we assessed whether GABA/GLx (ratio to NAA) levels in the mPFC/precuneus MRS voxels were associated with memory performance. Partial correlation analyses (Table 1) revealed no association between mPFC GABA levels and performance (*r* = −.216, *p* =.13) nor between mPFC GLx levels and performance (*r* =.223, *p* =.12). Neither, could we find an association between precuneus GABA levels (*r* =.08, *p* =.56) or precuneus GLx levels (*r* = −.105, *p* =.46) and performance. Second, we assessed whether neurotransmitter levels are associated with BMI. Similar, we could not find an association between GABA and BMI (mPFC; *r* =.132 *p* =.36/precuneus; *r* =.076, *p* =.59) nor GLx and BMI (mPFC; *r* =.122, *p* =.39/precuneus; *r* = −.204, *p* =.15). Third, we could not find an association between memory performance and BMI (*r* = −.138 *p* =.31).

**Table 1:** Depicted are the partial correlations controlled for age and gray matter ratio of the specific MRS Voxel

	Memory	BMI
ACC GABA	*r* = - .216 / *p* = .13	*r* = .132 / *p* = .36
ACC GLX	*r* = .233 / *p* = .12	*r *= .122 / *p* = .39
PRC GABA	*r* = .080 / *p* = .56	*r *= .076 / *p* = .59
PRC GLX	*r* = - .105 / *p* = .46	*r* = .076 / *p* = .59

### Neurotransmitter and memory performance (moderator analysis BMI)

**Fig. 6:**
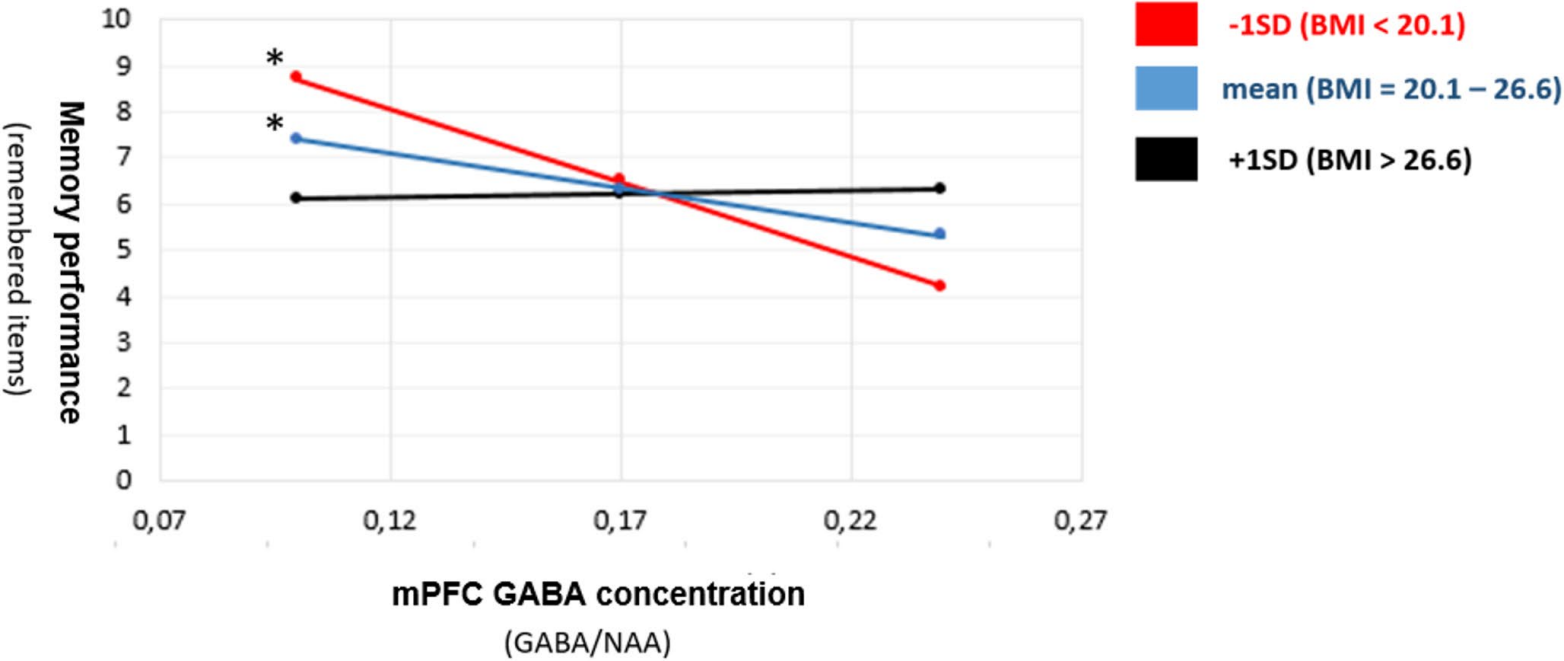
Depicted are the slopes of the three BMI categories regarding memory performance and GABA levels in the mPFC. Red is the group of Participants with a BMI below 20.1 (minus 1 standard division of the mean), blue the group with a BMI between 20.1 and 26.6 (mean) and black represents the group with a BMI above 26.1 (plus 1 standard division of the mean). The asterisk indicates the significant slopes

In a next step, a multiple regression model was computed to investigate whether there is an association between neurotransmitter levels and memory performance depending on BMI. We utilized the PROCESS macro for SPSS (PROCESS v2.16, Hayes, 2016) by using model 1 (moderation analysis with one continuous moderator variable) as implemented in the PROCESS macro. Regarding mPFC GLX (model summary *p* =.12/interaction *p* =.68), precuneus GABA (model summary *p* =.19/interaction *p* =.33) and precuneus GLx (model summary *p* =.24/interaction *p* =.66) we could not find a significant effect. However, we found a significant effect regarding mPFC GABA. This analysis revealed a significant model summary (*R* =.515, *p* <.025). The interaction between mPFC GABA and BMI was also significant (*B* = 5.17 SE = 2.5, *p* <.05), suggesting that the effect of mPFC GABA on memory performance depended on BMI. Simple slopes for the association between mPFC GABA level and memory performance were tested for low (−1 SD below the mean), moderate (mean), and high (+ 1 SD above the mean) levels of BMI. Two of the simple slope tests revealed significant negative associations between mPFC GABA levels and memory performance (Fig. 6). We found that mPFC GABA levels were significantly related to memory performance in participants with low BMI (B = −32.0, SE = 12.33, *p* <.013) and moderate BMI (B = −15.16, SE = 7.33, *p* <.05) but not high BMI (B = 1.66, SE = 9.39, *p* =.860). A one-way ANOVA was conducted to investigate if the memory performance of the three BMI groups (below 20.1, 20.1–26.6 and above 26.6) were statistically different. We found no significant difference between the groups(F(2,64) = 0.239, *p* =.788).

## Discussion

In the present study we aimed at delineating the relationship between BMI, GABA/glutamate and memory and found an association between mPFC GABA neurotransmitter levels and memory performance depending on BMI. Memory performance appeared to be predicted by mPFC GABA levels in individuals with a low and a moderate BMI but not in individuals with a BMI indicating overweight/obesity. Regarding mPFC Glx levels and precuneus GABA/Glx levels we did not find a moderating effect of BMI on the association between the neurotransmitters and memory performance.

It has long been known that the hippocampus is very important in episodic memory processes. However, there is increasing evidence that shows that coupling between the hippocampus and the mPFC, occurs at different stages of long-tefm memory formation. For instance, during encoding, neurons in the mPFC have been shown to exhibit activity that is phase locked to hippocampal activity (Siapas et al., 2005). Moreover, functional connectivity between this two regions, as measured with functional MRI (fMRI) in humans, has been shown to predict subsequent memory (Ranganath et al., 2005). It is assumed that he mPFC and the hippocampus may form a neural circuit for reactivation of memory traces that is crucial for the integration of novel information into neocortical networks (van Kesteren et al., 2010). Regarding the relation between neurotransmitter and memory it has been shown that performance in the face-occupation memory paradigm, as used in the present study, is negatively associated with mPFC GABA concentration (Thielen et al., 2019) and positively associated with mPFC GLx concentrations in humans (Thielen et al., 2018).

However, up to now, whether these effects are associated with BMI has not yet studied, even though there is growing evidence that a high BMI is associated with poor memory performance in humans (Lentoor and Myburgh 2022; De Wit et al., 2016; Clark et al., 2016; Gunstad et al., 2006). Unfortunately, the association between brain neurotransmitters and BMI in humans is less well studied. Actually, to the best of our knowledge, there is no study that assesses the relation between brain GABA concentration and BMI in humans. However, studies in rodents revealed that rats exposed to a high fat diet have altered GABAergic signaling in the hippocampus and PFC (Reichelt et al., 2015; Sandoval-Salazar et al., 2016). Moreover, these diet-evoked neuronal changes in GABAergic signaling were shown to manifest as cognitive deficits and dysregulated behavioral control in rats (Reichelt et al., 2015). This is in line with studies in humans showing that high fat or high sugar diet (Labouesse et al., 2016; Boitard et al., 2014; 2012; Holloway et al., 2011; Edwards et al., 2010) has pronounced and enduring detrimental effects on behavior, cognition and learning. In particular, memory tasks reliant on the hippocampus are rapidly disrupted by high sugar and high fat diets (Abbott, et al., 2016; Boitard et al., 2014; Francis & Stevenson 2013; Kanoski & Davidson, 2011; 2010; Kanoski, et al., 2007) and emerging data links consumption of high sugar and high fat diets to deficits in cognition facilitated by the PFC (Labouesse et al., 2016; Baker & Reichelt, 2016; Maayan et al., 2011).

Our findings indicate that a high BMI disrupts the association between mPFC GABA and memory performance and it suggests that overweight/obesity may change proper GABA signaling, which may be important in functional memory processes. While these disruption effects occurs only among those subjects with the highest BMI in our study, the effect does not seem to be linear, but may act in a more stepwise function. We can only speculate which mechanisms may be affected by overweight/obesity causing malfunctioning in memory related GABAergic processes. One possible explanation may be related to neuroinflammatory processes. Studies have shown that obesity is associated with increased neuroinflammatory processes in the brain (Clark et al., 2016; Reichelt et al., 2015; Miller and Spencer, 2014; Spencer, 2013). In this regard, abundant evidence supports the hypothesis that proinflammatory cytokines influence pathways involved in the regulation of mood and cognition, including neuroendocrine function, neurotransmitter metabolism and neural plasticity (Dinel et al., 2011). For instance, there is evidence that the release of proinflammatory cytokines as interleukin-1β suppresses GABA receptor activities at the postsynaptic site and reduces GABA synthesis at the presynaptic site (Wahab et al., 2019; Yan et al., 2015). Moreover, Samland and colleagues (2003) revealed that chronic interleukin 6 (IL-6) expression in the brain of mice results in progressive neurodegeneration which included GABAergic neurons. Hence, there is substantial evidence that several components of the GABAergic neurotransmitter system as for instance GABA synthesis and GABAergic neuronal density are altered by inflammation (see Crowley et al., 2016 for a review). Another possible explanation may be related to the engagement in physical activity. Since obesity is inversely related to physical inactivity (Friend et al., 2016) it may possible that obese people lack or have less neuronal processes that are triggered by the engagement in physical activation. For instance, it has been shown that exercise increases synaptophysin in mice which is associated with increased GABAergic signaling (Revilla et al., 2014) and that elimination of synaptophysin impaired object novelty recognition and reduced spatial learning in mice (Schmitt et al., 2009). However, one should note that this hypothesis on the role of engagement in physical activity does not exclude the hypothesis of neuroinflammation, since the engagement in physical activities is also associated with decreased neuro inflammation (Seo et al., 2019; Thielen et al., 2016; Spielman et al., 2016). Taken together, our finding may be related to changed cellular processes that may be caused through overweigt/obesity itself as for instance increased inflammation or caused through overweigt/obesity related behavior as physical inactivity or both.

Regarding GLx we could not find an association with memory performance nor BMI. A possible explanation may be the duration of the memory task used in the present study. In our previous work (Thielen et al., 2019), we utilized the same 4 min memory task and found an association between prefrontal GABA but not GLx levels and memory performance. However, as shown in Thielen and colleagues (2018), if the memory task takes around 22 min of time, GLx levels increased significantly and this increase predicted memory performance. Therefore, it may possible that the face-occupation memory paradigm may not be sensitive enough to GLx levels in the mPFC measured at a single time point, but rather to GLx reactivity or the change in the concentration, when excitatory neurotransmission is required over a longer period of time. In this regard, Stagg and colleagues showed that a higher concentration of GABA in the motor cortex of humans is associated with increased cortical excitability. The authors believed that this implausible relationship is driven by glutamate, since glutamate and GABA are metabolically linked; GABA is synthesized from glutamate via glutamate decarboxylase, and changes in one may therefore be reflected in the other (Stagg et al., 2011). Moreover, there are two major subtypes of GABA receptor within the cortex GABA-A receptors, a family of ligand-gated chloride channels and GABA-B receptors, metabotropic receptors linked to potassium channels (Stagg et al., 2011a). Stagg and colleagues (2011b) have demonstrated a relationship between GABA levels and 1ms SICI (a specific transcranial stimulation protocol), which they hypothesize may therefore reflect tonic extrasynaptic GABA-A activity.

Our results have to be interpreted in the presence of some limitations. First, we did not find a direct association between GABA levels and memory performance. This seems to be related to the group of subjects with overweight/obesity in our sample. If these individuals are excluded from the analysis there is a significant relation between ACC GABA and memory performance. Second, we did not find direct association between BMI and memory performance. A possible explanation may be the sample size. The effect of BMI on memory performance may be rather small since many factors as for instance lifestyle factors as engagement in physical activities (Thielen et al., 2016; Ruscheweyh et al., 2011) or intelligence (Oberauer et al., 2005) etc. have also an influence on memory performance. Thus, future studies should control for those variables to reduce possible influencing factors. Third, future studies may utilize a task with a prolonged learning phase, which may have an effect on glutamate concentrations in the mPFC (Thielen et al., 2018) associated with BMI and memory performance. Fourth, since the moderator analysis assess the significance of the indirect effects at differing levels of the moderator (mean BMI, minus 1 standard division of the mean and plus 1 standard division of the mean) the three BMI categories (below 20.1, 20.1–26.6 and above 26.6) do not align with definitions of overweight or obesity. The World Health Organization defines overweight with a BMI greater than or equal to 25 and obesity with a BMI greater than or equal to 30. Therefore, our finding does not distinguisch clearly between normal weight, overweight and obesity. Fifth, we did not adjust for multible comparisons. Therfore, the significant moderator effect of BMI on memory and mPFC GABA should be treated with caution. Sixth, in the present study we did not examine the hippocampus, even though this brain area is one of the most important in episodic memory processes. This was partly due to methodological issues and partly due to findings of previous studies in which we show that the brain areas selected in this study are involved in our memory paradigm (Thielen et al., 2015; Thielen et al., 2016; Thielen et al., 2018; Thielen et al., 2019). In particular, we found a change in GABA in the mPFC of patients with diabetes that was associated with memory performance (Thielen et al., 2019) which we aimed to investigate further in present study.

In summary, in our study we show that overweight/obesity, as measured with BMI, affects prefrontal GABAergic processes in a way that the relation between prefrontal GABA levels and memory performance disappears. This effect appeared to be absent in the posterior cortex which may indicate that prefrontal GABAergic processes are more affected through overweigt/obesity. Prefrontal GLx levels appeared not to be related to obesity nor memory performance which may be related to the memory task utilized. Future studies may have to replicate this finding and further assess the underlying mechanisms of our present finding.

## Supplementary Information

Below is the link to the electronic supplementary material.

Supplementary Material 1

## Data Availability

No datasets were generated or analysed during the current study.
