# Supplementary material for: The association between medial prefrontal GABA concentration and memory performance is disrupted in human with a high body mass index
Source: Brain Imaging Behav. 2026 Mar 27;20(2):64. doi: 10.1007/s11682-026-01121-1 (PMC13031246; doi:10.1007/s11682-026-01121-1)
Supplement: Supplementary file 1 — Supplementary Material 1 [file 11682_2026_1121_MOESM1_ESM.docx]

1. **Hardware**
2. Field strength 7 Tesla
3. Manufactorer Siemens Healthcare, Germany
4. Model Magnetom
5. RF coils single-channel transmit 32-channel head

coil (Nova Medical, Wilmington, MA)

1. Additional hardware N/A
2. **Acquisition**
   1. Pulse sequence MEGA-sLASER
   2. VOI locations mPFC and posterior cingulate gyrus
   3. VOI size 2x2x2cm^3^
   4. Repetition and echo time TR = 4,500 ms, TE = 80 ms
   5. Total number of excitations 64 [32 on, 32 off]
   6. Additional sequence parameters F1= 2500Hz; 1024 pts;

frequency offset -1.69 ppm (-507Hz)

- 1. Water suppression method WET
  2. Shimming method Automated B_0_ field mapping followed by

manual shimming of water < 30Hz

- 1. Triggering or motion correction N/A

1. **Data analysis methods and outputs**
   - - - 1. Analysis software AMARES / jMRUI software
         2. Processing steps Prior to fitting in jMRUI, spectra were

apodized with a 5 Hz Lorenzian filter, Zero filling to 512 points. Whereas, NAA was modeled from the “edit off spectra” as a single Lorentzian peak, GLx and GABA were modeled from the “different spectra” as a pair of Lorentzian peaks with the same line width as NAA

- - - - 1. Output measure Ratio to NAA
        2. Quantification references NAA was modeled from the “edit off

spectra” as a single Lorentzian peak at 2.01 PPM, GLx and GABA were modeled from the “different spectra” as a pair of Lorentzian peaks with the same line width (7.0 Hz) as NAA (GABA 2.97 und 3.06 PPM GLX 3.74 und 3.82)

1. **Data quality**

Reported variables SNR, linewidth

Data exclusion criteria No subjects excluded

Quality measure The SNR was calculated as the ratio of the

peak amplitude of **NAA** to the standard

deviation of the noise, measured in a

signal-free region of the spectrum (9–10

ppm)

Sample Spectrum Figure 3
